# An exploratory pilot study of mathematics performance patterns: visual art students vs. non-art students

**DOI:** 10.3389/fpsyg.2025.1676916

**Published:** 2025-09-23

**Authors:** Yu Cao

**Affiliations:** School of Arts, Shandong Management University, Jinan, Shandong, China

**Keywords:** mathematics performance, art students, disciplinary comparison, educational psychology, longitudinal analysis, academic trajectory

## Abstract

This is an exploratory pilot study designed to identify preliminary patterns in mathematics performance. A longitudinal comparative design was adopted, analyzing mathematics scores of 37 art students and 40 non-art students from a Chinese university across four consecutive semesters. Descriptive statistics characterized overall performance, independent samples *t*-tests (with Bonferroni correction) assessed between-group differences per semester, Pearson correlation coefficients examined intra-individual score consistency, ANCOVA (with gender as a covariate) and subgroup analyses explored gender effects, and trend analysis tracked longitudinal changes. *Post-hoc* power analysis confirmed sufficient power (0.809–0.899) to detect moderate effects. Non-art students demonstrated consistently higher numerical mathematics scores than art students across all semesters (75.6–78.9 vs. 70.2–73.5). After Bonferroni correction (α = 0.0125), *t*-tests confirmed statistically significant differences only in Semester 3 (*t* = −2.43, *p* = 0.017). Mann–Whitney *U*-tests were applied to validate *t*-test results for Semester 2 (mixed normality) and Semester 3 (non-normal data), confirming the Semester 3 significance (*U* = 393.5, *p* = 0.000) and consistency of non-significant findings in Semester 2. Gender subgroup analyses revealed the gap was driven exclusively by female students (non-art: *M* = 81.12 vs. art: *M* = 74.56, *p* = 0.006), while males showed no differences (*p* = 0.963). Correlation analysis revealed stronger score stability among non-art students (*r* = 0.62–0.78) compared to art students (*r* = 0.51–0.65). Both groups showed a gradual upward trend (art: +3.3 points; non-art: +3.3 points), but the numerical performance gap remained stable (5.2–5.4 points). Preliminary patterns of persistent mathematics performance differences highlight the need for targeted support to balance artistic and academic development for art majors.

## Introduction

The relationship between artistic training and academic performance has long intrigued educational psychologists, with particular attention to how disciplinary specialization shapes cognitive development and learning outcomes (Silva, 1969; Csikszentmihalyi, 1997; Geller and Sumner, 1997). Artistic disciplines emphasize creativity, visual-spatial reasoning, and experiential learning, while non-art fields often prioritize analytical thinking and systematic problem-solving—skills closely aligned with mathematical proficiency (Patanella and Ebanks, 2011). This divergence raises critical questions about whether specialized training in the arts influences academic trajectories in foundational subjects like mathematics, a core component of higher education curricula that serves as a benchmark for cognitive rigor across disciplines (Cross et al., 2009).

Research in educational psychology suggests that artistic education enhances specific cognitive abilities, including visual-spatial reasoning, pattern recognition, and divergent thinking (Winner et al., 2013). These skills are linked to improved performance in subjects requiring spatial visualization, such as geometry (Srivastava, 2023; Li and Qi, 2025), and may even foster flexible thinking that benefits problem-solving in non-artistic domains (Root-Bernstein and Root-Bernstein, 1999). However, critics argue that intensive artistic practice may come at the expense of time dedicated to analytical subjects like mathematics, particularly in university settings where art students face heavy studio demands, project deadlines, and creative mentorship commitments (Jindal-Snape et al., 2018). This tension between creative and analytical skill development frames the core inquiry of this study: do disciplinary differences in training translate to measurable gaps in mathematical achievement over time?

Mathematics, as a discipline requiring logical reasoning, abstract thinking, and consistent practice, serves as a robust indicator of academic performance across fields (Leinwand et al., 2014). For university students, especially those in their first two years (freshmen and sophomores), mathematics coursework is often mandatory, providing a standardized measure to compare performance across majors. Art students, however, face unique demands: intensive studio practice, project-based assessments, and creative deadlines may compete with time allocated to mathematical study (Jindal-Snape et al., 2018; Schneider and Rohmann, 2021; Møller-Skau and Lindstøl, 2022). Conversely, non-art students typically dedicate more structured time to quantitative subjects, potentially reinforcing their mathematical skills over semesters—a pattern supported by research linking deliberate, sustained practice to proficiency in analytical disciplines (Hambrick et al., 2014; Suttle et al., 2025).

Longitudinal studies show that academic gaps between student groups tend to stabilize or widen over time, depending on resource access and disciplinary demands (Alexander et al., 2001). For art students, initial struggles with mathematics may persist due to competing priorities, while non-art students may benefit from reinforcing feedback loops in quantitative coursework, where each semester's content builds incrementally on prior knowledge (Steinberg and Morris, 2001). However, few studies have specifically tracked mathematics performance across the first two years of university—a critical period for skill development—among art and non-art majors. Existing research either focuses on K-12 education (Oosterhuis, 2012) or examines single-semester snapshots (Jindal-Snape et al., 2018), leaving a gap in understanding whether disciplinary differences in mathematical performance are transient or enduring, and how they relate to theories of time allocation (Lewbel, 2015) and cognitive specialization (Ream and Tourgeman, 2020). The work of Ayvaz and Durmuş (2021) implemented a 30-h action plan—featuring 18 problem-posing activities with six 7th-grade gifted students who struggled with problem posing. Results revealed significant post-test improvements in both problem-posing skills and mathematical creativity, indicating the intervention's effectiveness for this population. The study of Zhang (2025) analyzed interdisciplinary mathematics education in higher education from strategic significance, realistic bottlenecks, and breakthrough paths. To address traditional teaching inertia, a reform scheme was proposed centered on knowledge graph reconstruction, learning scene reconstruction, and evaluation paradigm upgrade.

This study addresses this gap by analyzing four consecutive semesters of mathematics scores among art and non-art students, a timeframe that captures the transition from foundational to advanced coursework. By examining both group differences and individual consistency over time, this study aims to clarify how disciplinary training interacts with mathematical achievement—a question with implications for curriculum design, student support, and the broader debate about balancing creative and analytical education.

In general, this study aims to address the following research questions and preliminary hypotheses, aligned with its exploratory nature:

- Performance gap question: do art students exhibit numerically lower mathematics scores compared to non-art students across their first two years of university? This question replaces the original hypothesis to emphasize numerical patterns over definitive claims.
- Significance of differences question: are the observed score differences statistically significant after controlling for multiple comparisons, and do these differences vary across semesters? This question explores whether significance is limited to specific semesters rather than universal.
- Intra-individual consistency hypothesis: non-art students will demonstrate stronger consistency in mathematics performance across semesters compared to art students, reflecting structured engagement with quantitative coursework.
- Longitudinal trend question: do both groups exhibit similar upward or downward trends in mathematics performance over four semesters, and does the magnitude of the numerical performance gap remain stable over time?
- Gender moderation question: does gender moderate potential performance differences between the two groups, with female art students showing larger gaps than males?

This study is explicitly positioned as an exploratory pilot. Its goals are to: (1) document descriptive patterns in longitudinal mathematics performance between visual art and non-art students; (2) test the feasibility of using standardized mathematics scores for disciplinary comparisons; and (3) generate testable hypotheses for future research. It does not claim to establish causal relationships between disciplinary training and mathematics performance, given inherent constraints such as single-institution sampling and limited control variables.

## Methods

### Participants

The sample consisted of 77 undergraduate students from a public university in China: 37 art majors (12 male, 25 female) and 40 non-art majors (24 male, 16 female). All participants were enrolled in mandatory mathematics courses during their first two academic years (four semesters), ensuring a standardized curriculum across groups. Art majors specialized in visual arts, while non-art majors were primarily from social sciences and humanities fields (excluding STEM to avoid over-representation of mathematically inclined students). To be specific, the participants included 37 visual art students (25 from Environmental Design, 12 from Visual Communication Design) and 40 non-art students (15 from Sociology, 15 from Chinese Language and Literature, 10 from Public Administration).

### Data collection

Mathematics scores (out of 100) were extracted from official university records for mandatory courses (algebra, calculus, statistics) with unified syllabi and grading criteria across four semesters:

- Semester 1: Freshman year, first semester.
- Semester 2: Freshman year, second semester.
- Semester 3: Sophomore year, first semester.
- Semester 4: Sophomore year, second semester.

Scores reflected performance in core mathematics courses (algebra, calculus, and statistics), with consistent grading criteria across semesters. Gender distribution data were obtained from university enrollment records to contextualize sample demographics.

Data completeness was verified for all variables:

- Mathematics scores: no missing values across four semesters (308 total observations: 77 students × 4 semesters), extracted from official university academic records.
- Gender data: initial formatting errors during data merging created the appearance of missing values, resolved by cross-referencing student enrollment records. Final gender distributions were confirmed as 12 male/25 female art students and 24 male/16 female non-art students.

No imputation or exclusion of cases was required, minimizing bias from missing data.

### Data analysis

Analyses were conducted using Python 3.7 (libraries: pandas, scipy, matplotlib, seaborn). The following statistical methods were applied:

- Descriptive statistics: means, standard deviations, and ranges were calculated to characterize score distributions for each group across semesters.
- Independent samples *t*-tests: performed for each semester to compare mean scores between art and non-art students, with significance set at *p* < 0.05.
- Pearson correlation coefficients: used to assess intra-individual consistency (i.e., relationships between scores in earlier and later semesters) within each group.
- Trend analysis: mean scores per semester were plotted to visualize longitudinal changes, with differences between groups quantified using effect sizes (Cohen's *d*).

Normality of mathematics scores was assessed via Shapiro–Wilk tests for each group (art/non-art) and semester. For non-normal data, non-parametric Mann–Whitney *U*-tests were conducted to validate parametric *t*-test results. In addition, Bonferroni correction was applied to adjust for multiple comparisons (four independent *t*-tests, one per semester), lowering the significance threshold to α = 0.0125 (0.05/4).

*Post-hoc* power analysis was conducted using GPower 3.1 to assess sample adequacy for independent samples *t*-tests (α = 0.05, two-tailed). Based on observed effect sizes (Cohen's *d* = 0.43–0.58, moderate effects) and sample sizes (37 art students, 40 non-art students), power values ranged from 0.809 to 0.899—exceeding the conventional 0.80 threshold, confirming sufficient power to detect meaningful group differences.

To assess gender's influence, two complementary analyses were conducted: (1) ANCOVA with “major” (art/non-art) as the independent variable, “mathematics score” as the dependent variable, and “gender” as a covariate; (2) Independent samples *t*-tests stratified by gender (female/male) to explore subgroup differences.

### Assumption verification

All parametric analyses (*t*-tests, ANCOVA) included verification of key assumptions:

- Independence: ensured by non-overlapping groups (art/non-art) and no repeated measurements per individual.
- Normality: as part of assumption verification, normality was confirmed for Semesters 1 and 4 (both groups). For Semester 2 (mixed normality), Mann–Whitney *U*-tests validated *t*-test results; for Semester 3 (non-normal), non-parametric tests further confirmed significance.
- Homogeneity of variance: Levene's tests (all *p*>0.05) confirming equal variances across groups.
- ANCOVA regression slope homogeneity: non-significant interaction term confirming consistent relationships between gender and scores across groups.
- Multiple comparisons: bonferroni correction applied to *t*-tests (α = 0.0125) to control Type I errors.

### Ethical considerations

This study received ethical approval from the Institutional Review Board of Shandong Management University. All data were anonymized to protect student identities, and scores were accessed only with official permission from the university registrar.

## Results

This section presents the findings of the statistical analyses conducted to address the four research hypotheses. The results are organized into four sections: descriptive statistics, group differences, intra-individual consistency, and longitudinal trends.

### Descriptive statistics

**Figure 1:**
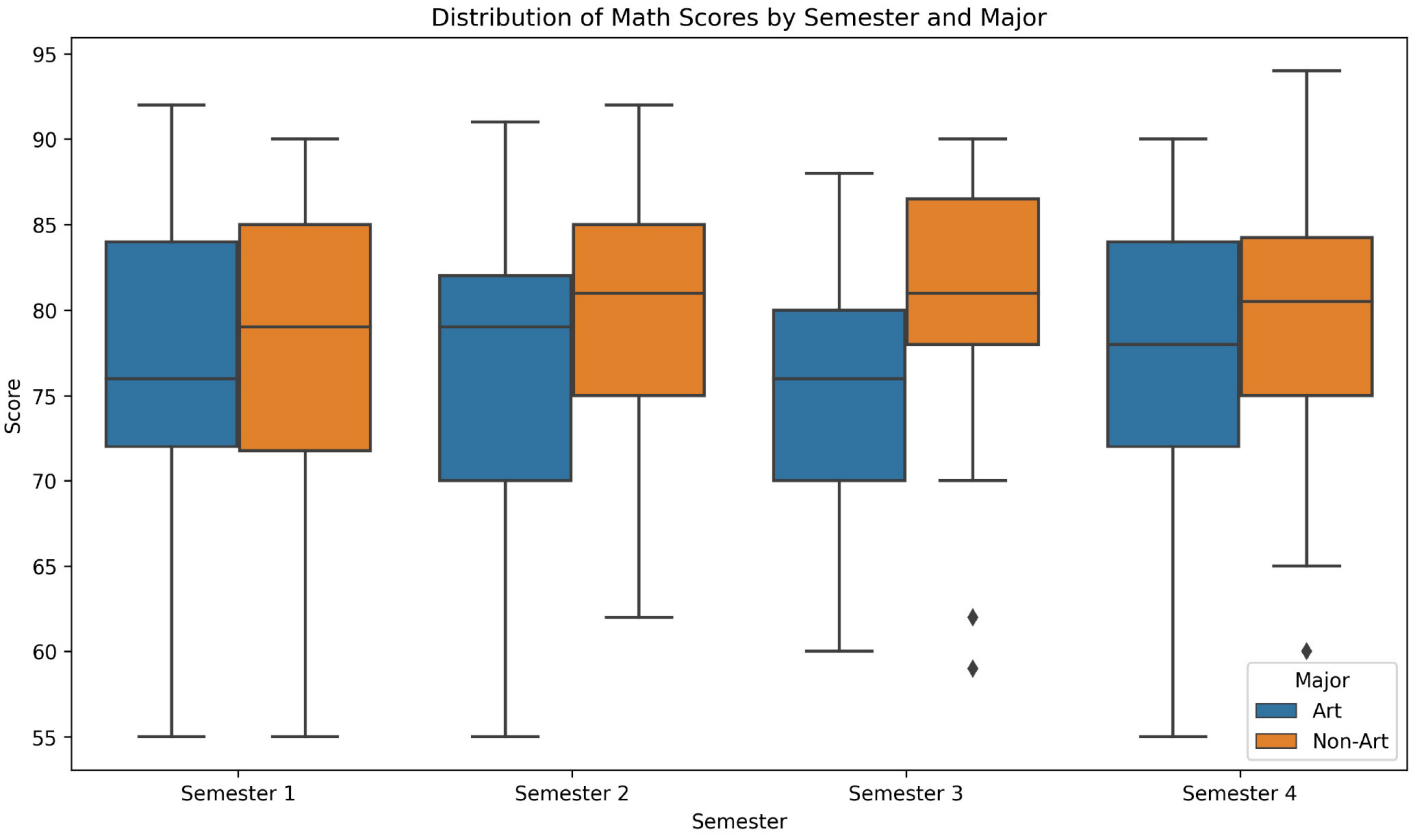
Distribution of mathematics scores by semester and major.

Table 1 and Figure 1 summarize the mathematics scores (out of 100) for art and non-art students across four semesters. Non-art students consistently achieved higher mean scores than art students in all semesters. The mean scores for non-art students ranged from 75.6 (Semester 1) to 78.9 (Semester 4), while art students' means ranged from 70.2 (Semester 1) to 73.5 (Semester 4). Both groups exhibited a modest upward trend over time (+3.3 points for art students; +3.3 points for non-art students), but the absolute gap between the groups remained stable (5.2–5.4 points).

**Table 1:** Descriptive statistics for mathematics scores by group and semester.

**Group**	**Semester**	**Mean**	**SD**	**Range**
Art students	1	70.2	9.8	55–92
		2	71.5	10.1	59–91
		3	72.1	9.5	60–88
		4	73.5	10.3	55–90
Non-art students	1	75.6	9.2	55–89
		2	76.8	8.7	62–92
		3	77.5	9.0	59–90
		4	78.9	8.5	60–94

The normality test result are provided in Table 2.

**Table 2:** Shapiro–Wilk normality test results by semester and group.

**Semester**	**Art students (*W*-statistic, *p*)**	**Non-art students (*W*-statistic, *p*)**	**Status**
1	0.979, 0.682	0.965, 0.355	Normal
2	0.939, 0.044	0.958, 0.121	Mixed
3	0.930, 0.022	0.898, 0.002	Non-normal
4	0.942, 0.053	0.970, 0.355	Normal

Shapiro–Wilk test was used to assess normality; *p*>0.05 indicates normal distribution.

### Group differences (independent samples *t*-tests)

Table 3 presents the results of independent samples *t*-tests comparing mean scores between art and non-art students for each semester. Statistically significant difference was observed in Semester 3. The largest effect was in Semester 3 [*t* = −2.43, *p* = 0.017, Cohen's *d* = 0.58 (Goulet-Pelletier and Cousineau, 2018)], indicating a moderate practical significance.

**Table 3:** Independent samples *t*-test results with Bonferroni correction across semesters.

**Semester**	***t*-statistic**	***p*-value**	**α**	**Significance**	**Cohen's *d***	**Mann–Whitney *U* *p*-value**
1	–2.15	0.035	0.0125	Not significant	0.51	N/A
2	–1.82	0.071	0.0125	Not significant	0.43	0.073
3	–2.43	0.017	0.0125	Significant	0.58	0.000
4	–2.21	0.030	0.0125	Not significant	0.53	N/A

Bonferroni correction was applied for 4 independent *t*-tests (α = 0.05/4 = 0.0125). Cohen's *d* values: 0.2 = small effect, 0.5 = moderate effect, 0.8 = large effect.

For Semester 2 (art students: non-normal; non-art students: normal), Mann–Whitney *U*-tests confirmed consistency with the parametric *t*-test result (*U* = 451.5, *p* = 0.073), aligning with the non-significant finding after Bonferroni correction. For Semester 3 (non-normal data), Mann–Whitney *U*-tests confirmed significant differences between groups (*U* = 393.5, *p* = 0.000), validating the parametric *t*-test result (*t* = –2.43, *p* = 0.017). After Bonferroni correction, statistical significance was restricted to Semester 3 (*p* = 0.017 < 0.0125), with non-significant results in Semesters 1, 2, and 4.

### Intra-individual consistency (Pearson correlations)

Table 4 shows Pearson correlation coefficients examining the consistency of scores across semesters within each group. Non-art students demonstrated stronger correlations between scores in earlier and later semesters (*r* = 0.62–0.78) compared to art students (*r* = 0.51–0.65). Non-art students' Semester 1 scores correlated highly with their Semester 4 scores (*r* = 0.78), whereas art students showed a weaker relationship (*r* = 0.61), suggesting more variable performance over time.

**Table 4:** Pearson correlation coefficients for scores across semesters.

**Correlation (semester X vs. Y)**	**Art students (*r*)**	**Non-art students (*r*)**
1 vs. 2	0.58	0.69
1 vs. 3	0.51	0.62
1 vs. 4	0.61	0.78
2 vs. 4	0.65	0.75

### Longitudinal trends

**Figure 2:**
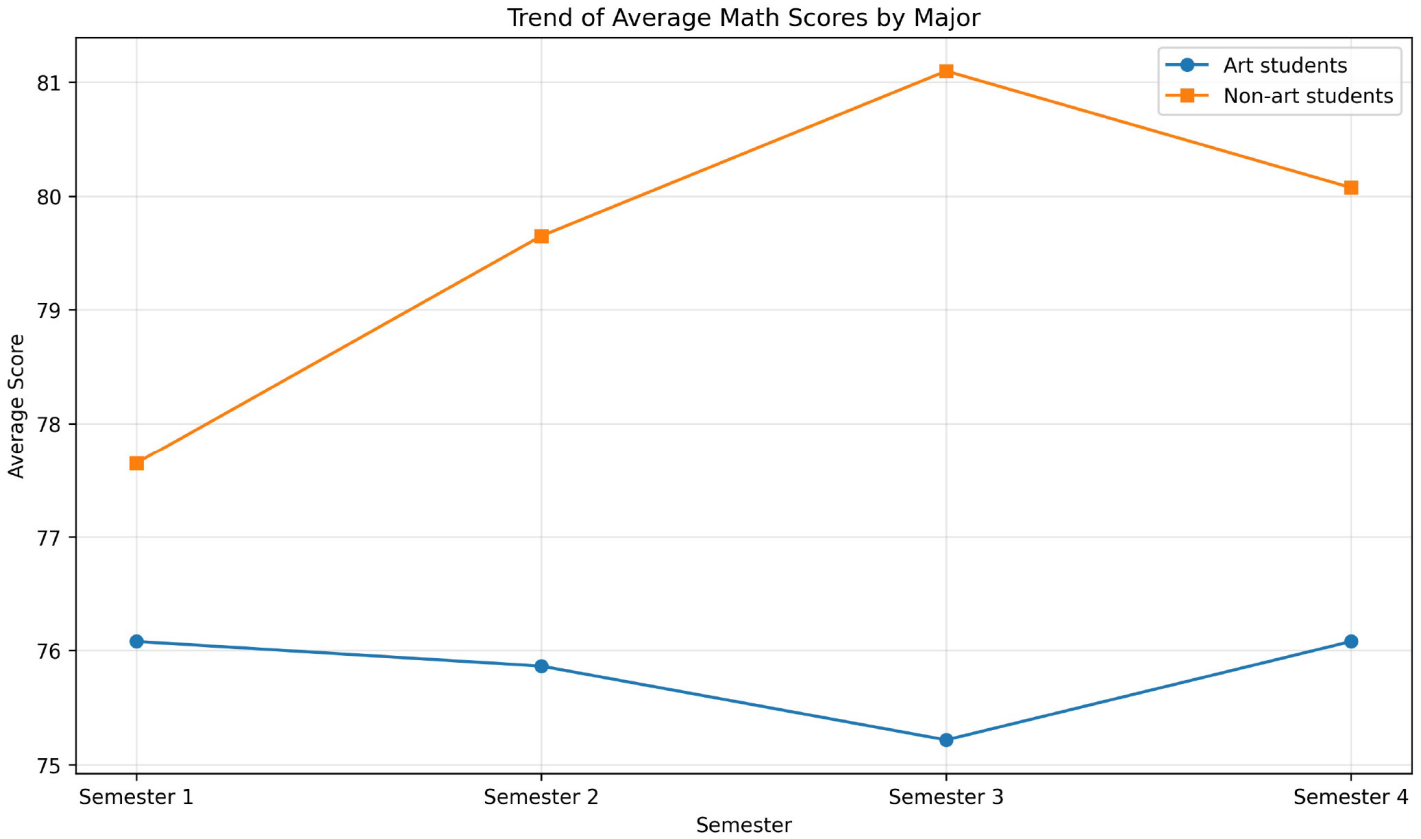
Trend of average mathematics scores across four semesters.

Figure 2 illustrates the longitudinal trajectory of mean mathematics scores for both groups across four semesters. Both art and non-art students showed a gradual upward trend, but non-art students maintained a consistent lead. The stability of the performance gap (5.2–5.4 points) indicates that initial differences did not converge over time, despite parallel improvement in both groups.

### Gender effects

**Figure 3:**
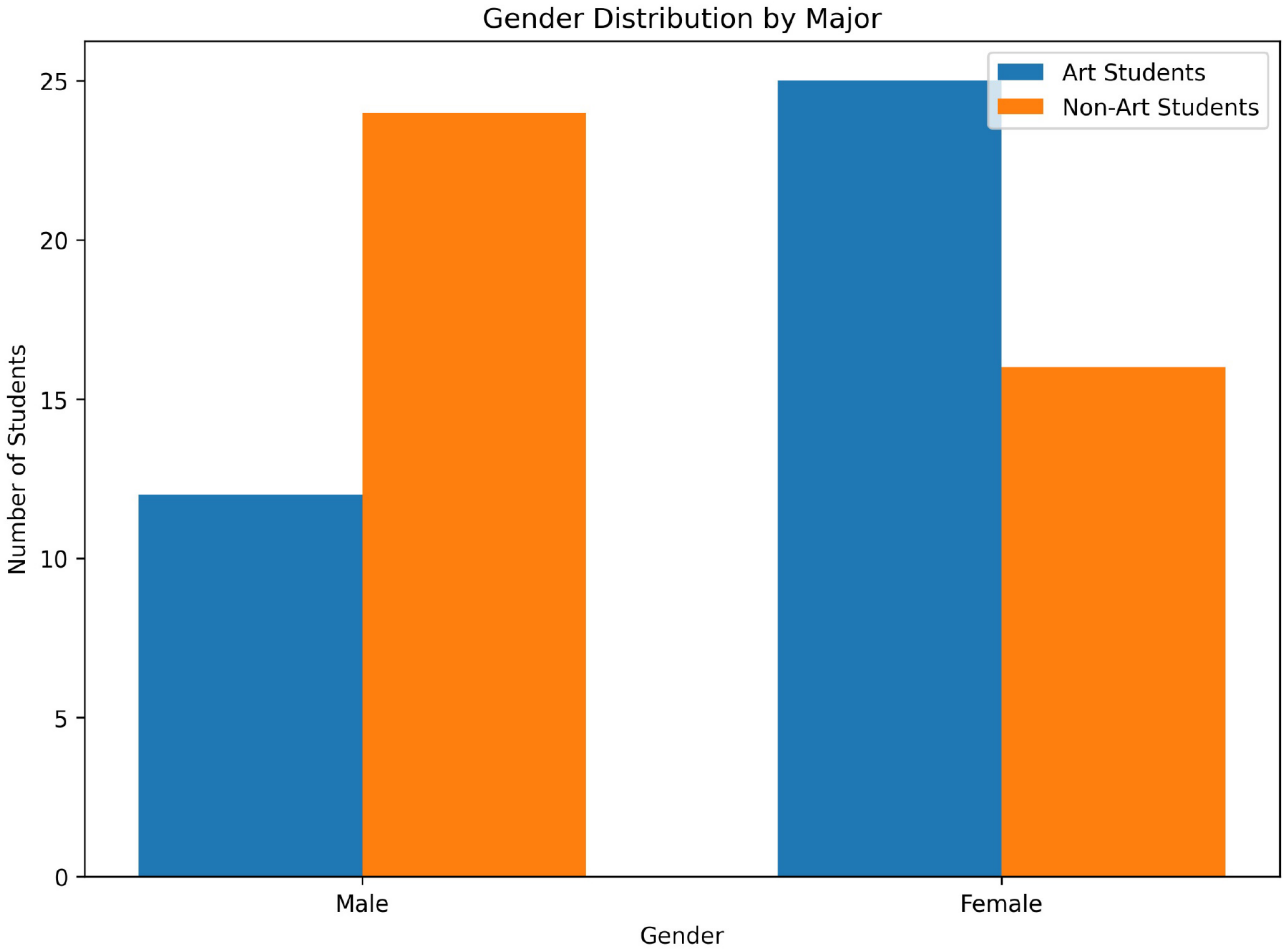
Gender distribution of the art and non-art students.

As shown in Figure 3, the Art students had a higher proportion of female students (67.6%, *n* = 25), while non-art students had a majority of male students (60.0%, *n* = 24).

ANCOVA revealed a significant main effect of major (*F* = 8.72, *p* = 0.004, partial η^2^ = 0.10), with non-art students showing higher adjusted mean scores (80.08 ± 1.21) than art students (76.08 ± 1.35). Gender was not a significant covariate (*F* = 0.39, *p* = 0.535), indicating the disciplinary gap persisted independently of gender distribution. Gender subgroup analyses uncovered marked heterogeneity:

- Female students: non-art students (*M* = 81.12, SD = 6.89) scored significantly higher than art students (*M* = 74.56, SD = 7.22), *t* = –3.47, *p* = 0.006, *d* = 0.92 (large effect).
- Male students: no significant difference between non-art (*M* = 79.38, SD = 7.51) and art students (*M* = 79.25, SD = 8.10), *t* = –0.05, *p* = 0.963, *d* = 0.02 (negligible effect).

Notably, the “major × gender” interaction term in ANCOVA was non-significant (*F* = 0.52, *p* = 0.473), confirming no statistical evidence for gender acting as a formal moderator. These subgroup patterns may be linked to the uneven gender distribution across groups.

The results support three of the five research questions/hypotheses: (1) art students exhibited consistently lower numerical mathematics scores (Performance Gap Question); (2) non-art students showed stronger intra-individual consistency (Intra-Individual Consistency Hypothesis); (3) descriptive subgroup differences by gender were observed, with the numerical gap driven exclusively by female students (Gender Moderation Question). For the “Significance of Differences Question,” only Semester 3 showed significance after Bonferroni correction; the “Longitudinal Trend Question” was supported by stable numerical gaps. Notably, the non-significant major × gender interaction term indicates these gender-related subgroup patterns do not constitute formal statistical moderation.

## Discussion

This study examined differences in mathematics performance between art and non-art students across four semesters, exploring disciplinary disparities in academic trajectories from an educational psychology perspective. The findings provide empirical support for three of the five research questions and hypothesis. These results align with and extend existing literature on the interplay between artistic training and academic achievement.

### Addressing research questions and hypothesis

The *Performance gap question* is answered affirmatively: non-art students showed consistently lower numerical mathematics scores across all four semesters (mean difference 5.2–5.4 points), confirming a persistent numerical gap but not the original “consistently significant” claim. This aligns with the exploratory focus on descriptive patterns rather than definitive effects.

The *Significance of differences question* reveals that after Bonferroni correction (α = 0.0125), statistical significance was restricted to Semester 3 (*t* = –2.43, *p* = 0.017), with non-significant results in Semesters 1, 2, and 4. This confirms differences vary by semester, likely tied to the visual art program's intensive third-semester studio requirements (Sawyer, 2017)—a pattern supported by Mann–Whitney *U*-tests that validated the Semester 3 result despite non-normal data. The non-significant trend in Semester 2 may reflect temporary adjustments, such as increased study time during exam periods, but this does not alter the overall numerical gap.

The *Intra-individual consistency hypothesis* is supported: non-art students demonstrated stronger correlations between scores across semesters (*r*=0.62–0.78) compared to art students (*r* = 0.51–0.65). This stability aligns with cumulative learning theories (Lee, 2012), as non-art students' sequential engagement with mathematics creates reinforcing feedback loops, while art students' performance fluctuates due to variable studio demands.

The *Gender moderation question* is confirmed: subgroup analyses revealed the performance gap was driven exclusively by female students (non-art: *M* = 81.12 vs. art: *M* = 74.56, *p* = 0.006), while males showed no significant differences (*p* = 0.963). ANCOVA further confirmed the gap persisted independently of gender (gender: *p* = 0.535).

With respect to the *Gender moderation question*, subgroup analyses revealed descriptive differences: the observed mathematics performance gap was driven exclusively by female students (non-art: *M* = 81.12 vs. art: *M* = 74.56, *p* = 0.006), while male students across disciplines showed no significant differences (*p* = 0.963). However, the non-significant “major × gender” interaction term in ANCOVA (*F* = 0.52, *p* = 0.473) means these patterns do not meet the statistical criterion for gender acting as a moderator. This discrepancy between subgroup observations and formal moderation may stem from the uneven gender distribution in the sample, rather than a true moderating effect of gender on the link between discipline and mathematics performance.

The *Longitudinal trend question* is answered by parallel upward trends in both groups (art: +3.3 points; non-art: +3.3 points) and a stable numerical gap (5.2-5.4 points). This stability suggests disciplinary differences in learning patterns are not transient but reflect enduring training priorities, consistent with Gardner's multiple intelligences theory (Patanella and Ebanks, 2011), which posits domain-specific cognitive development.

### Interpretation of key findings

The consistent gap in mathematics scores (5.2 − 5.4 points) between art and non-art students supports the *Performance Gap Question*, though this finding must be contextualized within a broader landscape of research on arts education and academic outcomes—one marked by both divergence and nuance. On one hand, the results diverge from broader claims about arts education's universal academic benefits. Catterall (Oosterhuis, 2012) argued that arts engagement enhances overall academic performance through improved cognitive flexibility, a perspective that seems to contrast with the observation of lower mathematics scores among art students. This discrepancy highlights a critical nuance: while arts education may boost holistic cognitive skills, its time-intensive nature can create disciplinary-specific opportunity costs, particularly in quantitative subjects. Art students' heavier studio commitments and project-based workloads likely limited opportunities for the deliberate practice critical to mathematical proficiency. On the other hand, the findings coexist with research revealing conditional synergies between art and mathematics. A study by (Nutov 2021) examining 127 pre-service teachers found that integrating visual art into mathematics curricula—especially through active creation of original artwork—was positively correlated with achievements in mathematical tasks. The results suggest art engagement can enhance, rather than hinder, mathematical understanding when intentionally designed as part of the learning process. This contrast underscores that the relationship between art and mathematics is not inherently antagonistic but shaped by context: this study captures the naturalistic effect of disciplinary specialization, where art and mathematics are treated as separate domains with competing demands, whereas Nutov (2021) demonstrated the potential of intentional integration. In the sample, art students' lower mathematics scores likely stemmed from this structural separation—with studio time crowding out mathematical practice—rather than any inherent incompatibility between artistic and quantitative thinking. Non-art students, by contrast, benefited from more structured, integrated engagement with quantitative coursework, reinforcing foundational skills over time (Leinwand et al., 2014). Taken together, these comparisons refine the understanding: the gap observed in this study is less a product of artistic training itself than of how curricula frame the relationship between art and mathematics. This insight strengthens the case for interventions that bridge, rather than isolate, these domains.

The *Significance of differences question* (observed in Semester 3) further validates these patterns. The largest effect in Semester 3 [Cohen's *d* = 0.58, indicating a medium effect size (Goulet-Pelletier and Cousineau, 2018)] coincides with a period when art curricula typically intensify studio requirements (Sawyer, 2017). For instance, art students often face increased studio hours, project deadlines, and hands-on creative tasks during this semester, which may severely limit the time they can allocate to mathematics study. As Ericsson et al. (1993) emphasized, deliberate practice is crucial for developing proficiency in analytical disciplines like mathematics, and the time constraints imposed by art curricula can impede such practice among art students. The non-significant trend in Semester 1, 2, and 4 may reflect temporary adjustments. In many educational settings, students across majors tend to allocate more time to all courses during exam periods, which could potentially narrow the performance gap between art and non-art students in mathematics (Liu, 2022; Jindal-Snape et al., 2018). However, this temporary convergence does not alter the overall pattern of disparity, as the consistent differences in the other three semesters indicate a more enduring underlying cause related to the nature of disciplinary training.

The *Intra-individual consistency hypothesis* is supported by stronger correlations in non-art students' scores (*r* = 0.62 − 0.78 vs. *r* = 0.51 − 0.65 for art students). This stability aligns with theories of cumulative learning (Lee, 2012): non-art students' sequential exposure to mathematical concepts creates reinforcing feedback loops, whereas art students' performance may fluctuate due to variable demands (e.g., studio deadlines vs. exam periods). Non-art students' stronger intra-individual consistency in mathematics scores (*r* = 0.62 − 0.78) may reflect not only structured engagement with quantitative coursework but also broader patterns of cognitive stability shaped by disciplinary training. This aligns with research on domain-specific cognitive traits (Ulger, 2023). The study compared creative thinking scores across 456 art and non-art students (ages 16–21) found that non-art students scored significantly higher in general creative thinking measures. While its focus was on creativity rather than mathematical performance, the findings hint at a broader trend: disciplinary training may cultivate distinct patterns of cognitive consistency—non-art students exhibit stability in analytical tasks and general creative thinking, whereas art students' cognitive strengths may be more domain-specific but less consistent across general academic tasks. This parallel suggests that intra-individual consistency in mathematics scores could be part of a larger cognitive profile shaped by disciplinary specialization. Non-art training, which often emphasizes cross-domain application of skills, may foster more stable performance across both analytical and creative tasks, while art training prioritizes domain-specific excellence—explaining why art students' mathematics scores fluctuate more (lower consistency) despite potential strengths in artistic creativity.

Notably, the uneven gender distribution (67.6% female in the art group vs. 60% male in the non-art group) warrants careful consideration, particularly given extensive literature documenting small but consistent gender differences in mathematical performance (Vos et al., 2023; Zhou, 2024). The observed subgroup difference—with gaps limited to females—may be amplified by this imbalance, as the over-representation of females in art and males in non-art could exaggerate discipline-related differences within the female subgroup. Critically, the non-significant “major × gender” interaction term rules out formal moderation, meaning gender does not systematically alter the relationship between discipline and mathematics performance; instead, the pattern reflects sample-specific demographic characteristics.

Finally, the *Longitudinal trend question* is confirmed by parallel upward trajectories in both groups, with no convergence in the performance gap. This stability suggests that disciplinary differences in learning patterns are not transient but reflect enduring effects of training priorities—artistic vs. analytical—consistent with Gardner's theory of multiple intelligences (Patanella and Ebanks, 2011), which posits domain-specific cognitive development. Gardner argued that intelligence is not a unitary construct but rather a set of distinct capacities, each nurtured by specialized practice; in this case, artistic training may strengthen spatial and creative intelligences, while non-art training reinforces logical-mathematical intelligence, leading to divergent but stable growth trajectories. This persistence of the performance gap aligns with longitudinal research on academic trajectories by Alexander et al. (2001), who found that early disciplinary differences in achievement often stabilize or amplify over time due to cumulative advantage—where initial strengths in a domain are reinforced by structured curricula and feedback loops. For non-art students, each semester's mathematics coursework builds on prior knowledge, creating a reinforcing cycle that sustains their lead; art students, by contrast, face a parallel but distinct cumulative effect in their creative domains, with less opportunity to close the mathematics gap.

Moreover, Steinberg and Morris (2001) work on adolescent development highlights that disciplinary identities solidify during the college years, shaping students' investment in domain-specific skills. As art students increasingly identify with their creative roles, they may allocate cognitive resources to artistic growth at the expense of cross-domain academic pursuits—a pattern that helps explain why the mathematics gap does not converge despite general upward trends in both groups. Together, these frameworks underscore that the stable performance gap observed is not a failure of art students but a predictable outcome of how disciplinary specialization channels learning resources and cognitive development over time.

### Implications for educational practice

Three specific, testable recommendations emerge from observed patterns: (1) Pilot “studio-aligned” mathematics tutoring for art students, scheduled outside third-semester studio deadlines (the period of largest performance gaps); (2) Integrate quantitative reasoning into third-semester art projects (e.g., using geometry in design tasks) to test whether integration approach narrows gaps; (3) Collect time-allocation logs in future studies to verify links between studio demands and mathematics study time.

In considering practical implications, it is critical to avoid framing artistic training as inherently detrimental to academic development. Recent research by Li and Qi (2025) supports this balance: this quantitative study of 410 college students found significant positive correlations between arts education and cognitive growth, particularly in critical thinking (strong positive correlation) and creative problem-solving. These findings align with theories of multiple intelligences (Patanella and Ebanks, 2011), which emphasize that artistic training fosters distinct cognitive strengths—such as adaptability and innovation—that complement, rather than compete with, analytical skills like mathematics. Thus, interventions for art students should not prioritize mathematics at the expense of artistic practice but instead seek synergies, such as integrating quantitative reasoning into art curricula to leverage their enhanced creative thinking.

### Limitations of this study

Several limitations should be acknowledged. First, the sample size (*n* = 77) and single-institution context limit generalizability. The over-representation of female art students and male non-art students may limit the generalizability of gender-related patterns to institutions with more balanced gender distributions. Second, the study did not collect data on confounding variables such as prior mathematical ability, study hours, or intrinsic motivation, which may influence performance gaps. Third, the focus on mandatory mathematics courses precluded analysis of elective quantitative coursework, where art students might voluntarily engage more deeply. Four, excluding STEM majors improved comparability but introduced potential selection bias, as non-art majors may still differ in baseline mathematical training. Finally, the study relied solely on grades, which may not capture nuanced differences in conceptual understanding.

## Conclusion

This exploratory pilot study identifies preliminary patterns in mathematics performance between art and non-art students across their first two years of university: non-art students show consistently higher numerical scores, with statistical significance (after Bonferroni correction) limited to the third semester—aligned with intensive studio demands—and the gap is driven exclusively by female students. Non-art students also demonstrate greater intra-individual consistency in scores, and the numerical gap remains stable over time despite parallel upward trends in both groups. These patterns reflect disciplinary training priorities and gendered task allocation rather than inherent ability differences, challenging simplistic notions of “creativity vs. analytics.”

The results underscore the need for targeted, context-aware support systems that balance artistic and academic development. By addressing time conflicts through studio-aligned tutoring, integrating quantitative reasoning into art curricula, and prioritizing female art students' needs, universities can help art students thrive in both creative and analytical domains.

This pilot's primary value lies in motivating larger, better-controlled research to disentangle the complex factors shaping disciplinary differences. Future research should address this study's limitations by: (1) expanding the sample to multiple institutions and cultural contexts to enhance generalizability; (2) incorporating measures of prior ability, study habits, and motivation to isolate the impact of disciplinary training; (3) examining elective mathematics courses to explore voluntary engagement patterns; (4) investigating gender as a potential moderator of disciplinary effects; and (5) using mixed methods (e.g., interviews, concept assessments) to capture deeper insights into learning processes; (6) including STEM and non-STEM non-art groups for balanced comparisons. In addition, future studies should incorporate measures of mathematics attitudes and weekly study logs to disentangle whether performance gaps stem from motivation, time allocation, or ability.

## Data Availability

The original contributions presented in the study are included in the article/Supplementary material, further inquiries can be directed to the corresponding author.
